# Arbuscular Mycorrhizal Fungi Enhance Sea Buckthorn Growth in Coal Mining Subsidence Areas in Northwest China

**DOI:** 10.4014/jmb.1907.07007

**Published:** 2020-03-13

**Authors:** Yanxu Zhang, Yinli Bi, Huihui Shen, Longjie Zhang

**Affiliations:** 1 State Key Laboratory of Coal Resources and Safe Mining, China University of Mining and Technology (Beijing), Beijing 00083, P.R. China; 2 College of Geoscience and Surveying Engineering, China University of Mining and Technology (Beijing), Beijing 100083, P.R. China

**Keywords:** Arbuscular mycorrhizal fungi, growth, mining subsidence, rhizosphere environment, sea buckthorn

## Abstract

Land subsidence induced by underground coal mining leads to severe ecological and environmental problems. Arbuscular mycorrhizal fungi (AMF) have the potential to improve plant growth and soil properties. We aimed to assess the effects of AMF on the growth and soil properties of sea buckthorn under field conditions at different reclamation times. Inoculation with AMF significantly promoted the survival rate of sea buckthorn over a 50-month period, while also increasing plant height after 14, 26, and 50 months. Crown width after 14 months and ground diameter after 50 months of inoculation treatment were significantly higher than in the uninoculated treatment. AMF inoculation significantly improved plant mycorrhizal colonization rate and promoted an increase in mycelial density in the rhizosphere soil. The pH and electrical conductivity of rhizosphere soil also increased after inoculation. Moreover, after 26 and 50 months the soil organic matter in the inoculation treatment was significantly higher than in the control. The number of inoculated soil rhizosphere microorganisms, as well as acid phosphatase activity, also increased. AMF inoculation may play an active role in promoting plant growth and improving soil quality in the long term and is conducive to the rapid ecological restoration of damaged mining areas.

## Introduction

Coal is the most important energy source in China, accounting for about 70% of the national primary energy consumption [1]. With the exhaustion of coal resources in east China, the proportion of coal resource exploitation in the west has gradually increased. In recent years more than 60% of coal output has been produced in Shanxi, Shaanxi, Inner Mongolia, and Ningxia provinces in west China [2]. However, most of the western region is arid or semi-arid and the ecosystem is very fragile. Mostly underground coal mining causes deformation and movement of strata and of the ground surface leading to a series of ecological problems including land subsidence, water resource disruption, mining waste disposal and air pollution [1, 3]. The mining subsidence induced by underground coal mining destroys soil structure, changes soil properties, and causes many environmental problems such as loss of soil moisture and nutrients, limitation of vegetation growth, death of plants and loss of soil microbial diversity and functioning [4-6]. Due to poor site conditions and deterioration of the environment, the function of the rhizosphere soil microbial community in the subsidence areas has declined, making plant establishment more difficult in these areas [7]. Beneficial microorganisms have been suggested as an effective and necessary tool for vegetation restoration in degraded mining areas [8].

Arbuscular mycorrhizal fungi (AMF) are common endophytic fungi that can form potentially symbiotic associations with more than 80% of terrestrial plants [9]. Mycorrhizal fungi live inside the cortex of plant roots, on the surface of the roots, or around the epidermal cells of the roots and the mycorrhizal symbiosis can affect plant growth and fitness [10]. It has been reported that AMF have the potential to increase the tolerance of their host plants to various stresses (*e.g.*, extreme temperatures, drought, salinity, and potentially toxic metals) [11-13]. AMF mycelium can increase the uptake of nutrients by plants and promote plant growth and the yield and quality of fruit [14, 15]. Moreover, AMF can restore or maintain soil fertility and reduce soil erosion [16, 17]. Based on these effects, AMF offer much benefit in the process of ecological restoration of reclaimed mining areas [18]. At present, many studies have applied AMF in mine reclamation and have achieved good effects [19-21]. However, most studies have been conducted under greenhouse conditions with sterilized soil and there are few reports on the effects of AMF on plant growth and the rhizosphere environment over a relatively long reclamation period under field conditions. Also, it is not clear whether the inoculation will work over a longer period of time. Hence, in our study we aimed to evaluate the role of arbuscular mycorrhizal inoculation on plant growth and soil properties over different reclamation periods of up to 50 months in coal mining subsidence areas.

## Materials and Methods

### Study Site Description

**Fig. 1:**
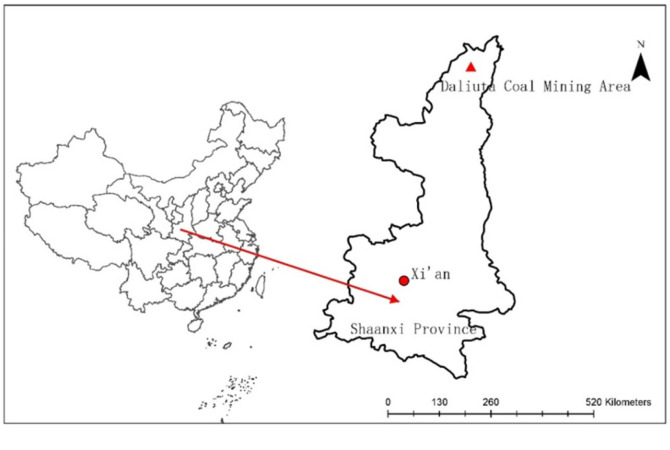
Map showing the location of the study area.

The experimental site is located in Shenmu County, Shaanxi Province, west China, at 38°50′N-39°47′N, 109°13′E-110°55′E (Fig. 1). This area is a combination of the northern Shaanxi plateau and Mu Us sandy land. The climate is typical mid-temperate continental monsoon with cold winters and hot summers, concentrated rainfall, and with large diurnal temperature differences. The annual average temperature is around 8.4°C, with a maximum of 38°C and a minimum of –28°C. The average annual precipitation is about 400 mm, and the average annual evaporation is 2,211 mm. The average altitude is about 1,300 m. The soil type is mainly aeolian soil, and the soil is relatively barren with a pH of 7.51, available P content of 2.30 mg/kg, available K of 126.4 mg/kg and a conductivity of 135.2 μs/cm.

### Materials and Field Experimental Design

The AMF strain used was *Funneliformis mosseae* (BGC XJ01, 1511C0001BGCAM0016) (abbreviated to F. m) supplied by Beijing Academy of Agriculture and Forestry Sciences. After propagation for 12 weeks on maize roots in pot culture, an inoculum was produced that consisted of maize root sections, mycorrhizal fungal spores and extraradical mycelium. The density of the mycelium was 4.6 m/g soil and the spore density was 65 spores/g soil. The test plant was sea buckthorn (*Hippophae rhamnoides* L.) which was obtained from a local nursery. The seedlings planted were about 20 cm high with consistent growth branches and undeveloped roots. In April 2012, the land was leveled and the seedlings were planted in the holes dug. The plant spacing was 2 m × 2 m, and inoculated (F.m) and uninoculated (CK) plants were set up. After the growth of the seedlings was stable the following July, 50 g AM fungal inoculum was inoculated near each sea buckthorn seedling root, as the inoculation treatment, while the control plot received equivalent sterilized AMF inoculum. Conventional management and protection measures were taken after the seedlings were inoculated.

### Sampling Methods

Plant growth was investigated on-site 2, 11, 14, 26 and 50 months after inoculation. The measured indexes were plant height, crown width, ground diameter and survival rate.

Two, 11, 14, 26, and 50 months after inoculation, sea buckthorn rhizosphere soil samples and fine root segments of sea buckthorn were collected from both inoculated and control treatments. Each treatment was replicated five times. The root segments collected were used to determine the mycorrhizal infection rate. The rhizosphere soil samples were placed in labeled sterile plastic bags and stored at 4°C and then transported to the laboratory as soon as possible. Soil samples were separated into two parts, one of which was sieved to 2 mm and stored at 4°C for analysis of soil enzyme activity and microbial counts and the other was air-dried and sieved to 1 mm for determination of soil physical and chemical properties and extra-radical mycelium density.

### Quantification of Mycorrhizal Colonization Rate and Hyphal Density

Mycorrhizal root colonization was evaluated by clearing the fine roots in 10% KOH and staining with 0.05% (w/v) Trypan blue in lacto-glycerol [22]. Root mycorrhizal colonization was assessed by the glass slide method [23]. The percentage of root mycorrhizal colonization was calculated by dividing the number of colonized roots by the total number of root samples examined.

The extral-radical mycelium was extracted using a modification of the aqueous membrane filtration technique [24]. Microfiltration membrane filters (0.45 μm pore size) containing the extracted hyphae were transferred to microscope slides and stained with two drops of 0.05% (w/v) Trypan blue in lacto-glycerol. Twenty-five visual fields were observed at 100 × magnification under a compound microscope and the mycelium density was calculated by counting the intersections using the grid cross method [25].

### Analysis of Soil Properties

Soil phosphatase activity was determined using the improved method of Zhao and Jiang [26]. The sodium bicarbonate-extractable P colorimetric method was used for soil available phosphorus. Soil organic matter content was determined by external heating of potassium dichromate. Soil pH value and conductivity were determined by pH meter (soil : water ratio 2.5:1) and conductivity meter (soil : water ratio 5:1), respectively [27].

The numbers of soil microbial groups were counted using the dilution plate method [28]. The bacteria were isolated after three days of incubation at a constant temperature of 28°C on beef extract peptone medium containing beef extract (0.3%), peptone (1%) and agar (1.8%) in deionized water, pH 7.0-7.2; actinomycetes were cultured for four days at a constant temperature of 28°C on modified Gause’s No. 1 synthetic medium containing soluble starch 2 g, KNO_3_ 0.1 g, K_2_HPO_4_ 0.5 g, MgSO_4_·7H_2_O 0.05 g, NaCl 0.05 g, FeSO_4_·7H_2_O 0.001 g, agar 1.8 g, deionized water 100 ml, pH 7.4-7.6; and fungi were incubated for two days at a constant temperature of 28°C on Martin-Bangladesh red medium containing Rose Bengal agar 3.5 g , deionized water 100 ml, natural pH. Numbers of bacteria, actinomycetes and fungi were recorded as colony forming units (CFU)/g of dry soil.

### Statistical Analysis

All data were subjected to analysis of variance (ANOVA) using the SAS software package (8.0) at the 5%significance level. Microsoft Excel 2013 and Origin 2017 were used to process the data and figures, respectively.

## Results

### Plant Growth and Survival Rate

At different reclamation times the plant height, crown width and ground diameter were higher than those in the uninoculated treatment (Table 1). Compared with the uninoculated treatment, the plant height of five monitoring periods increased by 5.4%, 14.4%, 53.9%, 24.2%, and 16.2%, respectively, and the crown diameter by 12.3%, 26.4%, 78.7%, 19.1%, and 13.0%. The ground diameter at different reclamation times increased by 4.2%, 6.4%, 8.1%, 12.1%, and 23.3% compared with the uninoculated treatment. The results show that inoculation with mycorrhizal fungi promoted the aboveground growth of sea buckthorn. Analysis of variance shows that AMF inoculation significantly increased plant height after 14, 26, and 50 months of inoculation but crown diameter showed a significant difference only at the 14th month. The ground diameter showed a significant difference 50 months after inoculation.

**Fig. 2:**
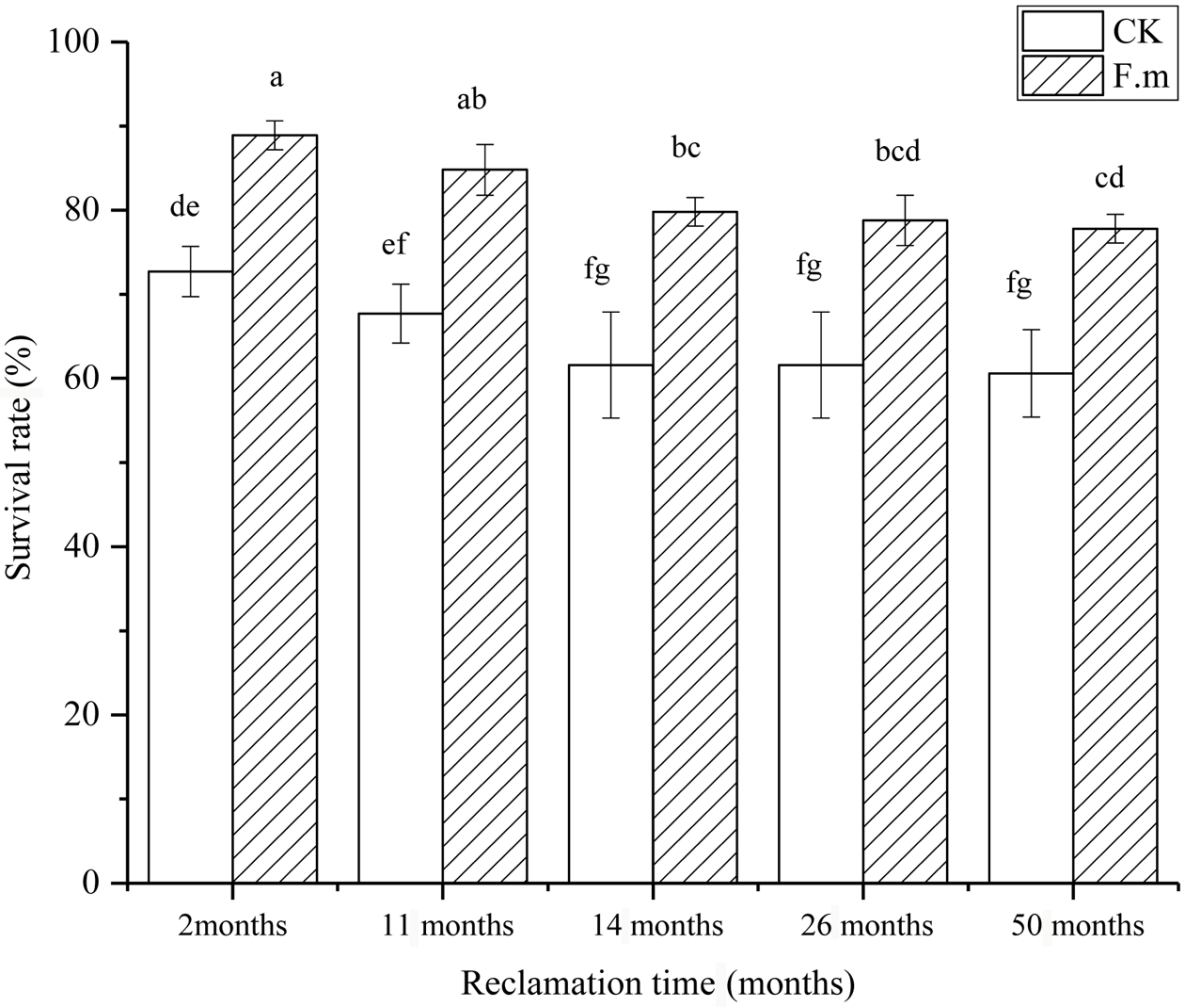
Effects of AMF on the survival rate of sea buckthorn in different reclamation years.

As shown in Fig. 2, two months after inoculation the survival rates of inoculated and control plants were 88.9%and 72.7%, respectively. The survival rate of inoculated plants increased by 22.3%. At the 11th and 14th months after inoculation the survival rate increased by 25.2% and 29.5%, respectively. With increasing reclamation time, the survival rate of both treatments decreased, but the survival rate of the inoculated plants was higher than that of the controls, indicating that AMF inoculation effectively increased the survival rate of the plants.

### Mycorrhizal Characteristics

**Fig. 3:**
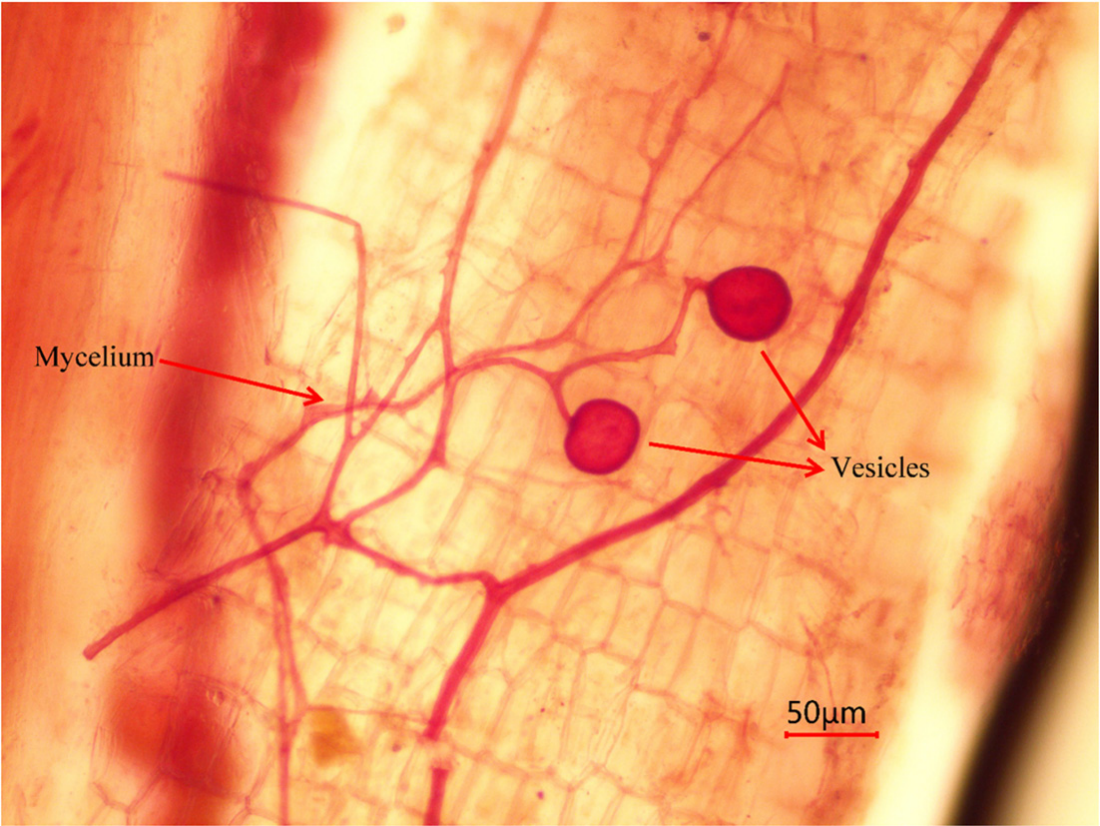
Vesicles and mycelium structures of AMF in root of sea buckthorn.

**Fig. 4:**
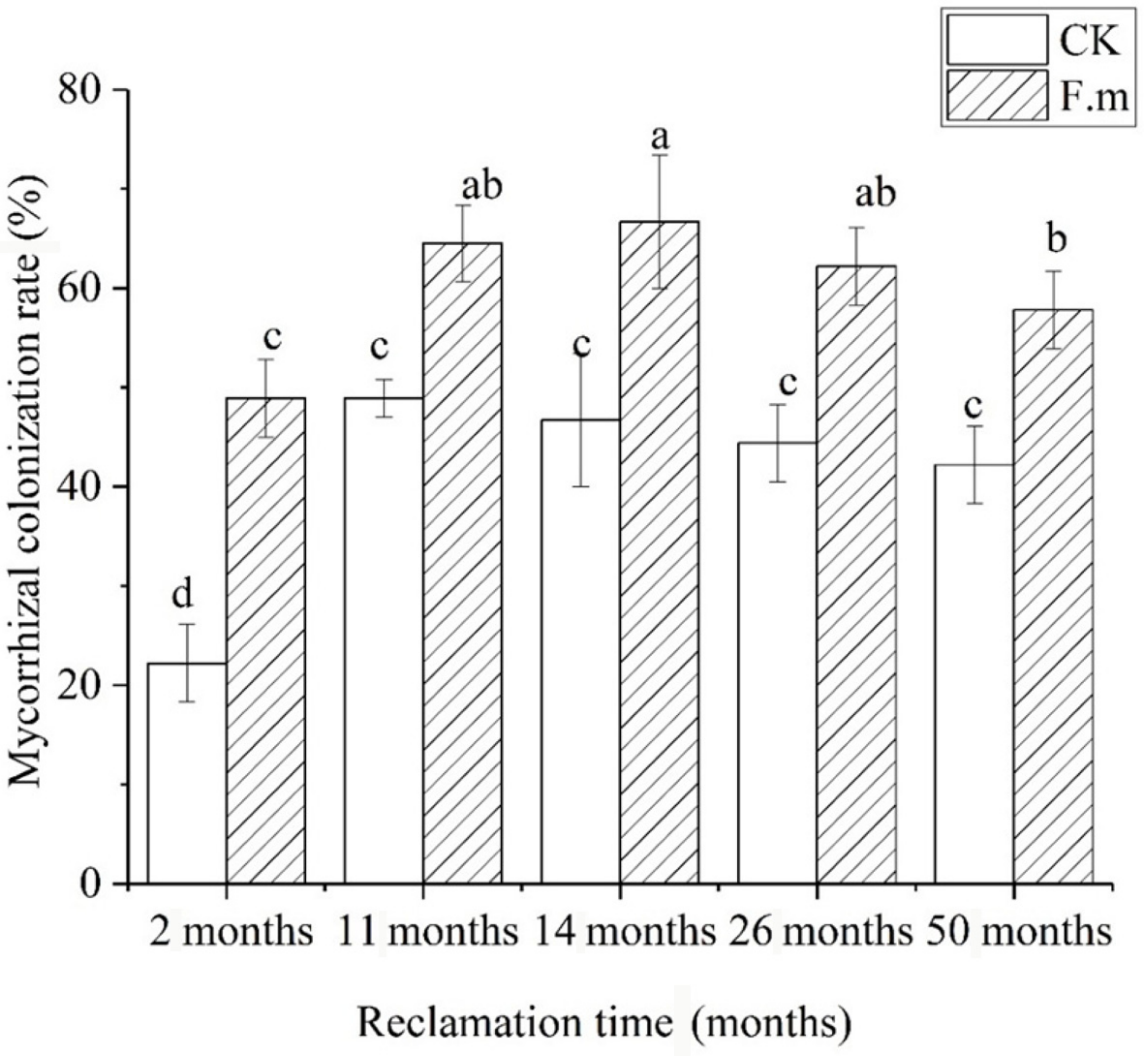
Mycorrhizal colonization rate of sea buckthorn in different reclamation years.

After AMF entered the cells of the plant root cortex, the mycelium could develop into arbuscular, vesicle, root mycelium or root spore structure under appropriate conditions [10]. In Fig. 3, vesicles and mycelium could be observed, which is considered to be the evidence of mycorrhizae presence, and it could be preliminarily proved that the root system of sea buckthorn was infected by mycorrhizal fungi. As shown in Fig. 4, the mycorrhizal colonization rate of the inoculated plants was significantly (40%) higher than that of the controls after two months, indicating that AMF formed a good symbiotic relationship with the root system. With increasing reclamation time, the mycorrhizal colonization rate of the inoculated treatment increased further and was always higher than that of the uninoculated control.

**Fig. 5:**
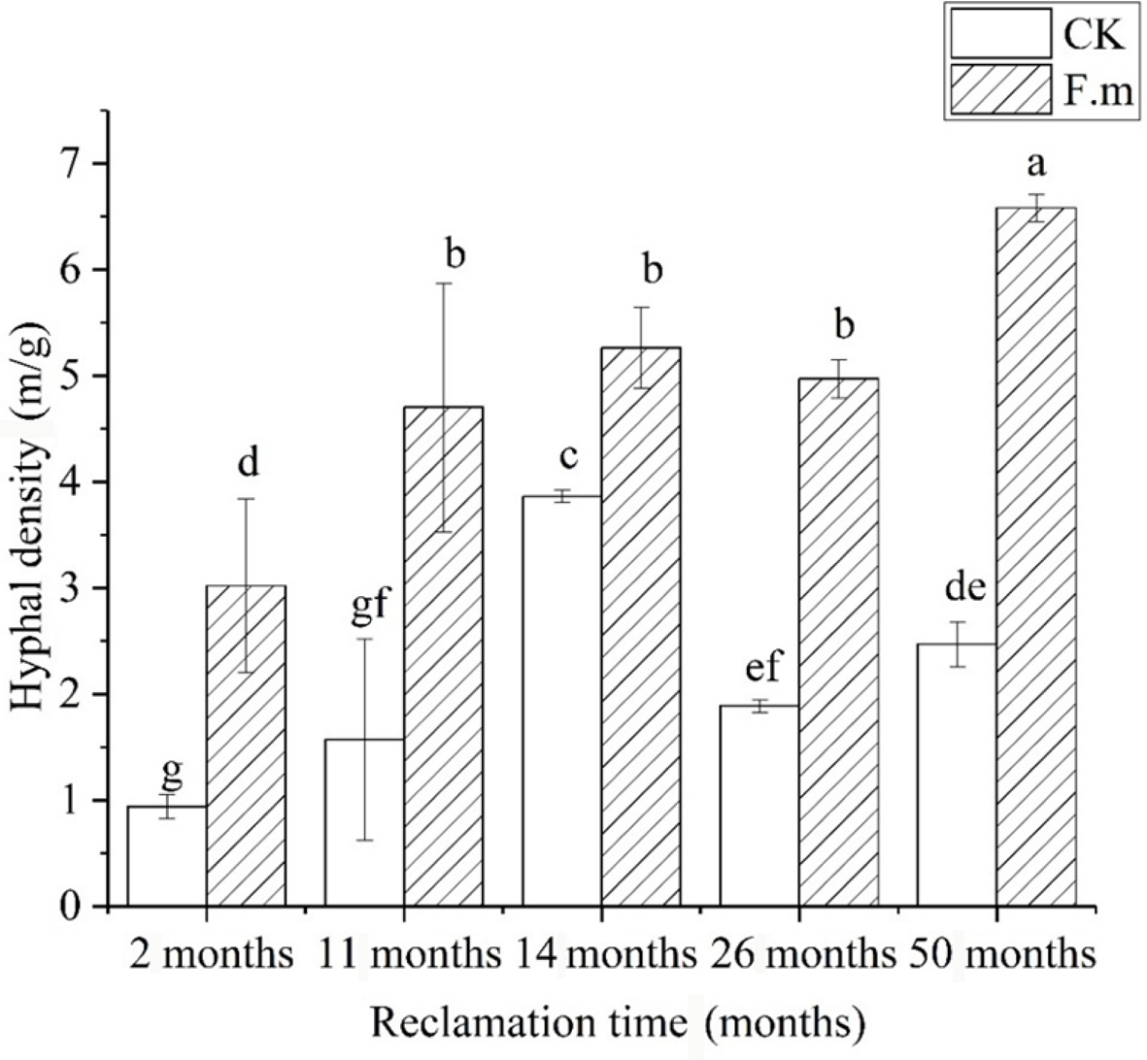
Hyphal density of sea buckthorn in different reclamation years.

The density of mycelia can be reflected by secretion levels and overall higher activity, indicating the greater effects on mycorrhizal plants. Two months after the inoculation, the inoculated treatment and control were 3.02 m/g and 0.94 m/g, respectively (Fig. 5). The inoculation significantly increased the length of soil mycelium, which was 221% higher than the control. At 11 and 14 months after inoculation, the inoculated treatment was significantly larger than the uninoculated control treatment. The increase in mycelium can expand the absorption area of the root system and increase the amount of water and nutrients taken up by plants from the rhizosphere soil through the mycelium, thus promoting plant growth.

### Soil Characteristics

The rhizosphere soil pH increased after the seedlings were planted and the pH in the mycorrhizal inoculation treatment was higher than that in the control, with significant differences between inoculated and control treatments after 2, 14 and 26 months (*p* < 0.05). After planting the soil, conductivity showed a downward trend, possibly due to the fast plant growth rate and uptake of a large number of metal cations from the soil. The soil conductivity in the mycorrhizal inoculated treatment was higher than in the control, with significant differences after 11, 26 and 50 months (*p* < 0.05). This may be attributed to the mycelium absorbing and storing nutrients after the formation of mycorrhizal structures (Table 2).

Changes in available nutrients in the rhizosphere during different reclamation periods were complex. F. m increased the content of soil available P compared with the control two months after inoculation. From 11 to 26 months after inoculation, available P in the soil was lower than in the control. Fifty months after inoculation the content of available phosphorus in the rhizosphere soil was significantly higher than that in the control (*p* < 0.05). This may have been due to a deficiency of phosphorus in the rhizosphere soil due to the large amount of nutrients required for early plant growth, while with increasing reclamation time and the action of mycorrhizal fungi the activities of rhizosphere acid enzymes were enhanced and the conversion efficiency of soil phosphorus increased.

The soil organic carbon content showed a decreasing trend within 14 months of inoculation, possibly due to stimulation of the rhizosphere microbial community by AMF and increased rhizosphere bacterial activity. Increasing reclamation time led to an increase in soil organic carbon content because of large numbers of dead plant branches and leaves as well as glomalin-related soil proteins (GRSP) produced by the AM fungi [29].

### Soil Microorganisms and Enzyme Activities

Soil microorganisms are important participants in the decomposition of organic matter and the cycling and metabolism of nutrients in soil, which are important soil quality evaluation indexes [30]. Two months after inoculation, the numbers of soil bacteria, fungi and actinomycetes increased significantly, reaching 139.3%, 31.9% and 28.8%, respectively, compared with the control. The increase in the soil microbial population may be due to an increase in rhizosphere secretion and the growth of plant root system. As the reclamation time increased the numbers of soil bacteria, fungi and actinomycetes in the inoculated treatment were higher than in the control. Analysis of variance shows that the number of fungi in the inoculation treatment was significantly greater than in the control at all reclamation times investigated (*p* < 0.05), and the number of actinomycetes was significantly higher except after two months of reclamation.

Soil enzyme activities are sensitive to mycorrhizal inoculation and can be used as biological indices of soil quality to evaluate the restoration process of degraded ecosystems. After inoculation with AMF, the development of the root systems accelerated and root exudation increased, promoting the physiology and metabolism of the root system. There was no effect after two months, but subsequently the soil acid phosphatase activity in the inoculation treatment was higher than in the control. The acid phosphatase activity increased by 10.0%, 13.2%, 39.4%, and 8.3%, respectively, compared with the non-inoculation treatment after 11, 14, 26, and 50 months. Analysis of variance shows that there were no significant differences after 2, 11, and 14 months but after 26 months the inoculation treatment was significantly higher by 39.4% over the control. This indicates that the enzyme activity increased significantly with increasing reclamation time after a functional symbiosis was formed between the mycorrhizal fungi and the root system.

## Discussion

Coal mining causes large-scale surface subsidence, cracking of the soil surface, decline in soil water and fertilizer conservation capacity, and the survival and growth of local vegetation are also affected by certain adverse environmental stresses. Studies have shown that AMF can effectively increase the stress resistance of sea buckthorn and contribute to the survival of the plants under adverse conditions in sterilized soil [31, 32]. Here, inoculation significantly increased the survival rate of the plants and promoted their growth. This may be related to the large amount of mycelium produced after the formation of mycorrhizal structures which promote the uptake of water and nutrients from the soil [10]. Our results are comparable to those of Jaroszewska *et al*. [33]. The survival and growth of vegetation can effectively increase the vegetation cover in western mining areas, and this is important in achieving successful revegetation and control of soil loss and erosion [17, 34].

Soil disturbance has been reported to significantly reduce the density of spores and the length of extra-radical mycelium of AMF (AMF populations) compared with undisturbed soil [35]. Coal mining subsidence greatly disturbs the soil and reduces the length of extra-radical mycelium of AMF, which will have negative effects on the formation of mycorrhizas in roots [36]. There were also a large number of mycorrhizal fungi in the soil at our study area as indicated by the root colonization rate of the sea buckthorn in the control treatment and in previous studies [37]. The mycorrhizal infection rate in roots was low after cessation of coal mining and inoculation with mycorrhizal fungi increased mycorrhizal infection and mycelial density, indicating that AMF inoculation can promote AMF propagules in disturbed soils, a result consistent with Caravaca [38]. The mycorrhizal infection rate and mycelial density were still higher than those of the uninoculated control after 50 months, indicating that AMF inoculation formed a good symbiosis with the host plant over a long time period. This has very important implications for the reclamation of fragile damaged mining areas.

The microbial community plays an important role in plant growth and improvement of soil quality and is also an important indicator for soil quality evaluation. Previous studies indicate that an increase in microbial quantity and diversity in soils can reflect soil quality, and the increase in soil microbial activity has a positive effect on plant growth, root activity and rhizosphere soil metabolism [39]. Zhao *et al*. suggested that with an increasing number of years of reclamation the quantity of microorganisms in the soil increases greatly, and soil quality also improves significantly [40]. This may be due to the fact that the root exudates produced by plants after reclamation provide more carbohydrates for microorganisms to promote the metabolic activities of rhizosphere microorganisms. Studies show that Frankensis (a type of actinomycete) plays a key role in the nitrogen fixation ability of sea buckthorn, and Tian *et al*. found that inoculation with mycorrhizal fungi enhanced the nitrogen fixation ability of sea buckthorn. Inoculation with mycorrhiza may have promoted the development of the actinomycete Frankia and improved nitrogen fixation ability [32]. Huang *et al*. showed that inoculation with mycorrhizal fungi significantly increased the number of fungi in soil in which white clover was growing but had little effect on the number of bacteria in the soil, while Li *et al*. found that AMF increased the numbers of bacteria and actinomycetes and reduced the number of fungi [41, 42]. Here, AMF improved the rhizosphere microenvironment, nutrient status and organic matter content, together with the acid phosphatase activity, which may have contributed to the increase in the number of microorganisms and in the metabolic activity of the rhizosphere. Unfortunately, the rhizosphere soil microbial community structure in AMF-inoculated and control treatments were not examined. Previous studies showed that AMF had a significant effect on the rhizosphere bacterial community and could significantly increase the bacterial diversity index in sea buckthorn root system, which indicated that AMF inoculation may affect microbial community composition [43-45]. Changes in soil microbial community structure merit further study.

At present, there are many successful examples of mycorrhizal application in field reclamation [46-48]. However, not all reports are positive. For example, a field study conducted by Stahl *et al*. showed that AMF inoculation produced no significant difference, which the authors attributed mainly to the ecological adaptability of mycorrhiza [49]. Kohler *et al*. reported that a combination of compost addition and arbuscular mycorrhizal inoculation rather than the separate use of each of these exerted effects on the growth of shrub species [50]. In addition, some studies show that indigenous AMF give the maximum effect [51]. Therefore, in order to ensure the success of reclamation, a series of factors need to be considered in the process of mycorrhizal microbial reclamation, such as the production and quality control of microbial agents in large-scale microbial reclamation, species compatibility with the target environment (*e.g.*, edaphic and climatic conditions in the reclamation area), the degree of spatial competition with other soil organisms in the target niche, host plant species and cultivars, and the timing of inoculation [52, 53]. Furthermore, small-scale field experiments are needed before large-scale mycorrhizal reclamation is attempted.

## Figures and Tables

**Table 1:** Effects of AMF on the growth of sea buckthorn in different reclamation years.

Reclamation time/month	Treatment	Height/cm	Crown width/cm	Ground diameter/cm
2	CK	27.61 ± 2.18e	22.83 ± 3.35e	5.14 ± 0.36g
	F.m	29.11 ± 2.55e	25.60 ± 3.16e	5.24 ± 0.40g
11	CK	28.50 ± 0.98e	23.06 ± 1.23e	6.57 ± 0.89gf
	F.m	32.62 ± 2.06e	29.22 ± 3.34de	6.99 ± 0.31ef
14	CK	36.91 ± 2.36e	26.83 ± 4.67e	7.90 ± 1.80df
	F.m	56.76 ± 2.51d	47.91 ± 1.20cd	8.54 ± 1.09de
26	CK	55.60 ± 6.54d	59.32 ± 3.75bc	9.34 ± 0.97cd
	F.m	69.06 ± 9.46c	70.63 ± 3.56b	10.47 ± 0.73c
50	CK	141.00 ± 20.87b	153.20 ± 16.54a	12.73 ± 1.07b
	F.m	163.80 ± 15.04a	173.0 ± 45.92a	15.70 ± 0.95a

Data are expressed as mean ± SD of five replicates, and different letters in each variable denote significant difference (*p* < 0.05) by least significant difference.

**Table 2:** Effects of AMF on the soil properties of sea buckthorn in different reclamation years.

Reclamation time/month	Treatment	pH	Ec (µs/cm)	SOC (g/kg)	AP (mg/g)
2	CK	7.81 ± 0.19d	135.28 ± 4.4a	17.53 ± 1.89a	2.43 ± 0.29c
	F. m	8.07 ± 0.07bc	140.96 ± 6.0a	10.96 ± 1.73de	3.58 ± 0.26a
11	CK	8.07 ± 0.15bc	107.06 ± 2.5cd	13.50 ± 3.85bc	2.71 ± 0.07b
	F. m	8.12 ± 0.10abc	114.66 ± 3.9b	9.33 ± 1.84ef	2.02 ± 0.10d
14	CK	8.05 ± 0.11bc	61.36 ± 4.2ef	13.31 ± 2.40bcd	2.04 ± 0.08d
	F. m	8.25 ± 0.13a	76.90 ± 12.5e	8.33 ± 1.83f	1.87 ± 0.32d
26	CK	8.03 ± 0.13c	95.32 ± 22.1d	8.10 ± 0.41f	2.04 ± 0.19d
	F. m	8.19 ± 0.03ab	111.78 ± 14.7bc	13.67 ± 0.19bc	1.83 ± 0.10de
50	CK	8.02 ± 0.08c	48.22 ± 1.5f	11.91 ± 0.19cd	1.61 ± 0.11e
	F. m	8.03 ± 0.07c	98.38 ± 24.6cd	15.36 ± 0.57ab	1.90 ± 0.25d

Ec, electrical conductivity; SOC, soil organic carbon; AP, available phosphorus.

**Table 3:** Effects of AMF on soil microorganisms and enzyme activity of sea buckthorn in different reclamation years.

Reclamation time/ month	Treatment	Bacteria/ (10^6^ CFU/g)	Fungi/ (10^4^ CFU/g)	Actinomyces/ (10^5^ CFU/g)	Phosphatase activity/ (mg/g)
2	CK	5.09 ± 0.80ef	9.69 ± 0.32b	4.32 ± 0.26gh	1.78 ± 0.20f
	F. m	12.18 ± 1.01d	12.79 ± 1.02a	5.56 ± 0.29fg	1.71 ± 0.11f
11	CK	16.07 ± 0.86c	3.94 ± 0.45de	16.07 ± 0.86b	4.91 ± 0.07bc
	F. m	18.69 ± 1.20b	7.13 ± 1.23c	18.69 ± 1.20a	5.40 ± 0.04ab
14	CK	5.46 ± 0.23ef	0.74 ± 0.33g	11.77 ± 1.08d	3.16 ± 0.07e
	F. m	6.09 ± 0.25ef	4.47 ± 0.26de	13.33 ± 0.26c	3.58 ± 0.16de
26	CK	4.47 ± 2.59f	1.57 ± 0.45g	3.33 ± 1.45h	4.36 ± 1.73cd
	F. m	11.09 ± 2.03d	3.25 ± 0.67f	6.85 ± 0.58f	6.08 ± 0.93a
50	CK	6.62 ± 1.53e	3.78 ± 1.69ef	4.44 ± 2.22gh	5.53 ± 0.32ab
	F. m	28.38 ± 1.89a	5.00 ± 0.84d	8.62 ± 1.21e	5.99 ± 0.38a
